# Monocyte chemoattractant protein-1 promoter polymorphism and plasma levels in alzheimer’s disease

**DOI:** 10.1186/1742-4933-10-6

**Published:** 2013-02-21

**Authors:** Elisa Porcellini, Manuela Ianni, Ilaria Carbone, Massimo Franceschi, Federico Licastro

**Affiliations:** 1 DIMES, School of Medicine, University of Bologna, Via S. Giacomo 14, Bologna, 40126, Italy; 2 Department of Neurology, IRCCS Multimedica, Milan, Italy

**Keywords:** Alzheimer’s disease, MCP-1 promoter polymorphism, MCP-1 plasma levels, MCI –AD conversion

## Abstract

**Background:**

Neurodegenerative disorders such Alzheimer's disease (AD) are often characterized by senile plaques and neurofibrillary tangle. In addition, reactive astrogliosis, microglia activation and a chronic inflammation are found in AD brain. Activated microglia has been reported to express a large number of beta chemokines including monocyte chemoattractant protein-1 (MCP-1). The potential role of MCP-1 in AD pathogenesis is supported by the over expression of MCP-1 associated with an increase of amyloid deposition in transgenic mice. MCP-1 protein may be regulated by a single nucleotide polymorphism (SNP) occurring at position −2518 of the MCP-1 gene promoter. In this paper we correlated the A-2518G MCP-1 SNP distribution in three different populations: AD, control and MCI (mild cognitive impairment) population to evaluate whether this SNP might be a risk factor for AD or for MCI-AD conversion. MCP-1 plasma levels were also measured and correlated to the cognitive impairment (CIND) and AD risk.

**Results:**

No differences in genotype distribution and allele frequencies of A-2518G MCP-1 SNP among AD patients, MCI subjects and controls were observed even after APOEe4 variation adjustment with logistic regression. However in MCI subjects, followed up for two years, this SNP appears to influence the progression of the disease; being the G allele slightly more frequent in MCI patients that developed AD. MCP-1 plasma levels were different among CIND (cognitive impairment but no dementia), AD and controls. The MCP-1 A-2518G promoter polymorphism did not affect MCP-1 plasma levels within the three populations.

**Conclusions:**

MCP-1 G allele did not affect the risk of AD, but slightly influenced MCI conversion to AD and MCP-1 plasma levels were increased in subjects with preclinical AD.

## Background

Alzheimer’s disease (AD) is the most common form of adult-onset dementia. Pathological hallmarks of AD include amyloid deposits rich in β-amyloid (Aβ) peptide, neurofibrillary tangles composed of hyperphosphorylated tau and the occurrence of activated microglia [1].

Etiopathogenetic mechanisms associated with age-related cognitive decline and AD are complex and still largely unclear. However, both genetic and environmental factors are implicated in the pathogenesis of the disease [2-5].

A substantial literature has suggested the involvement of several processes in AD onset, such as inflammation, plaque deposition, oxidative stress, cholesterol homeostasis and endothelial dysfunction [6-9].

A number of inflammatory mediators associated with amyloid deposits such as Interleukin-1 (IL-1), IL-6, transforming growth factor (TGF-β), TNF-α, acute phase proteins for instance α−1 antichymotrypisn (ACT) and α_2_-macroglobulin, and several complement factors have been detected in AD brains [10,11].

Inflammatory cytokines are produced by activated microglia and astrocytes and these molecules are able to stimulate the phagocitic activity of microglia [12]. Activated microglia has been reported to express a large number of beta chemokines including monocyte chemoattractant protein-1 (MCP-1) [13,14].

MCP-1, produced by neurons and glial cells, is a chemokine that, by regulating monocyte chemotaxis and endothelial activation, modulates inflammatory processes. The potential role of MCP-1 in AD pathogenesis is supported by the over expression of MCP-1 associated with an increase of Abeta deposition in Amyloid precursor protein (APP) transgenic mice [15]. In addition higher MCP-1 levels in cerebro spinal fluid (CSF) of AD patients than matched controls have been reported [16]. This latter finding has been confirmed by an independent recent study showing that CSF levels of MCP-1 were significant increased in subjects with AD compared to healthy controls [17]. Moreover, a recent study showed that elevated MCP-1 CSF levels were associated with an higher annual decrease of MMSE score in AD patients; these findings suggested that elevated CSF MCP-1 was associated with an accelerated rate of cognitive decline [18].

Data regarding MCP-1 plasma levels are conflicting. Several Authors showed that MCP-1 levels were elevated in AD patients or subjects with mild cognitive impairment (MCI) [19,20]. These data were not confirmed by other studies reporting no association between MCP-1 plasma levels and AD [12,21].

It has been shown that serum levels and biological activity of the MCP-1 protein may be regulated by a single nucleotide polymorphism (SNP) occurring at position −2518 of the MCP-1 gene promoter [22,23]. Several studied described this SNP as a risk factor for AD but results are not always concordant being this polymorphism differentially distributed in different populations [21,24-27].

Here we present data on MCP-1 A-2518G polymorphism in AD patients, in subjects with MCI and in a healthy elderly control population and its association with the risk of AD or MCI.

MCP-1 plasma levels were also measured in AD patients, subjects with cognitive impairment but no dementia (CIND) and healthy elderly followed up for five years and levels of MCP-1 were correlated with cognitive deterioration.

## Results

**Figure 1:**
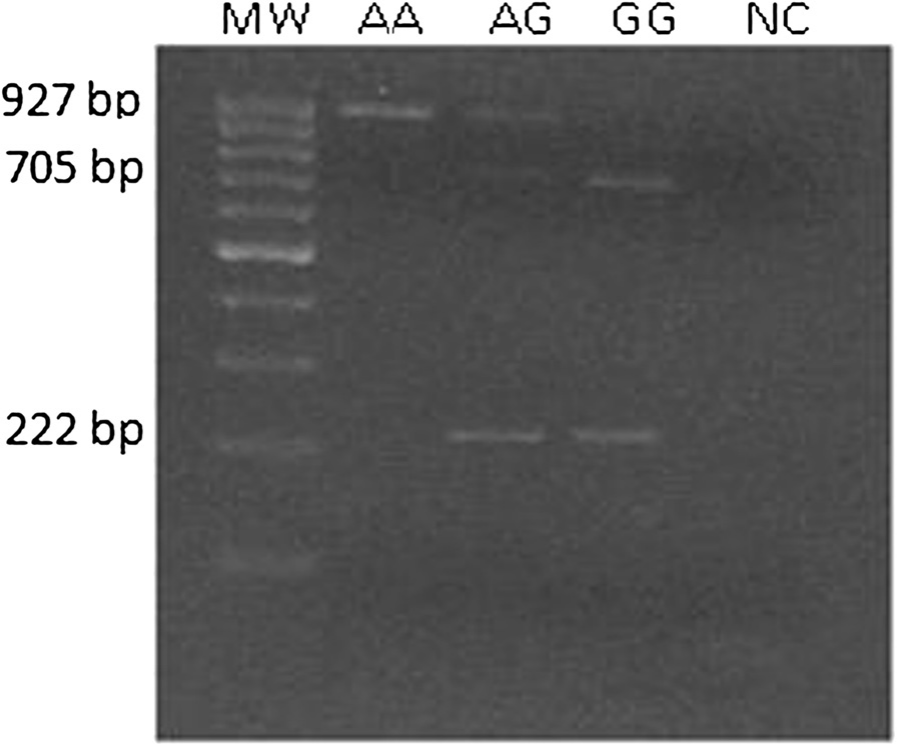
**MCP-1 A-2518G promoter polymorphism (rs699947) pattern after O/N digestion with PvuII enzyme.** Digestion resolved three different band patterns, according to the three different genotypes; e.g. at 222 bp and 705 bp (GG genotype), 222 bp, 705 bp and 927 bp (GA genotype) or only 927 bp (AA genotype). 100 bp Molecular weight (MW) and Negative control (NC) were also indicated in agarose gel.

As reported in Table 1, no difference in genotype distribution and allele frequencies of A-2518G MCP-1 SNP among AD patients, MCI subjects and controls (CTR) were observed (AD vs CTR χ2= 0.11, p=0.994; CTR vs MCI χ2= 3.633, p=0.163; AD vs MCI χ2= 3.942, p=0.139). An agarose gel with the three different pattern of bands (representing the three different genotypes) was showed in Figure 1.

**Table 1:** Genotype distribution and allele frequency of the A-2518G MCP-1 promoter polymorphism in patients with probable AD, patients with MCI and controls (CTR) from Northern Italy

	**AA**	**AG**	**GG**	**Allele A**	**Allele G**
	(n) %	(n) %	(n) %	(n) %	(n) %
AD (n=291)	(139) 47.8	(130) 44.7	(22) 7.6	(269) 92.4	(152) 52.2
MCI(n=88)	(49) 55.7	(37) 42.0	(2) 2.3	(86) 97.7	(38) 43.2
CTR(n=146)	(69) 47.3	(66) 45.2	(11) 7.5	(135) 92.5	(77) 52.7

Statistics.

AD vs CTR (genotypic distribution): Pearson χ^2^= 0.11, p=0.994;

logistic regression adjusted for age and APOE e4 p=0.713 c.i 95% (0.981-1.013).

CTR vs MCI (genotypic distribution): Pearson χ^2^= 3.633, p=0.163;

logistic regression adjusted for age and APOE e4 p=0.988 c.i 95% (0.970-1.007).

AD vs MCI (genotypic distribution): Pearson χ^2^= 3.942, p=0.139;

logistic regression adjusted for age and APOE e4 p=0.299 c.i 95% (0.994-1.018).

Logistic regression analysis regarding MCP-1 genotype distribution adjusted for age and APOEe4 was applied to these populations and results were not statistically significant; therefore age and APOEε4 presence did not affect MCP-1 genotype distribution (AD vs CTR p=0.713, AD vs MCI p=0.299, MCI vs CTR p=0.988) and MCP-1 genotype was not an independent risk factor for AD or MCI.

MCI patients were followed up for 2 years and cognitive performances were detected at the end of the study. Among the MCI group, 38 subjects evolved to AD, and 45 did not. Genotype distribution and allele frequency of MCI patients converting to AD compared to those remaining MCI were described in Table 2. The G allele was slightly more frequent in MCI patients that converted to AD after 2 years than in those remaining MCI, however the difference was not statistically significant (χ^2^ = 2.117, p= 0.337). This difference was not statistically significant also after logistic regression adjusted for age and APOEε4 presence (p=0.137).

**Table 2:** Genotype distribution and allele frequency of MCI patients converting to AD compared to those remaining MCI

	**AA (n) %**	**AG (n) %**	**GG (n) %**	**Allele A (n) %**	**Allele G (n) %**
MCI->AD (n=38)	(19) 50.0	(18) 47.4	(1) 2.4	(37) 97.4	(19) 47.4
MCI= MCI (n=45)	(28) 62.2	(17) 37.8	(0) 0	(45) 100	(17) 37.8

Pearson χ^2^ = 2.117, p= 0.337.

Logistic regression analysis adjusted for Age and APOE e4 : G allele p=0.137 c.i 95% (0.948-1.007).

**Figure 2:**
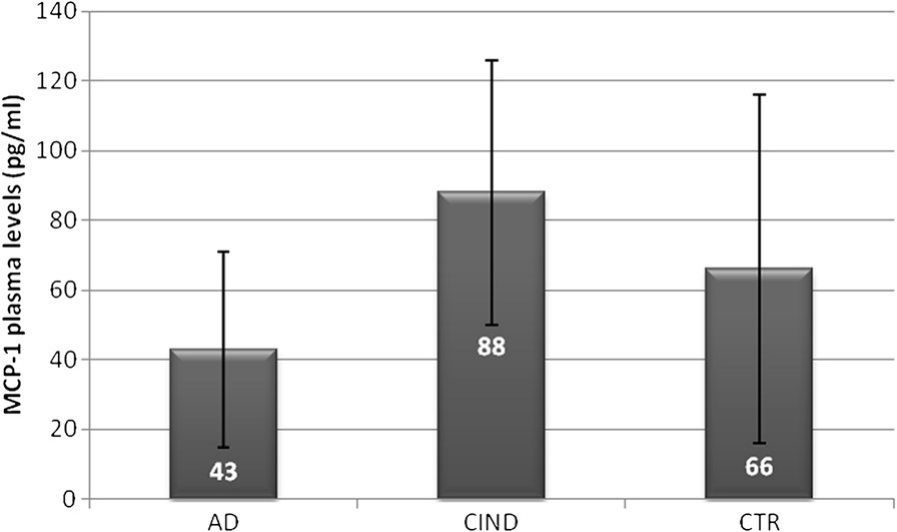
**MCP-1 plasma levels (pg/ml) in patient with AD, CIND and Controls.** ANOVA, F=15.695 p=0.0001

MCP-1 plasma levels were also detected in AD patients (n=87), controls (n=34) and CIND subjects (n=24), as shown in Figure 2.

AD patients showed lower MCP-1 levels (43 pg/ml ± 28 ) than CIND (88 pg/ml ± 38) and controls (66 pg/ml ± 50), differences in MCP-1 levels among the three groups were statistically significant and elevated in CIND (ANOVA test F= 15.695 p=0.0001; Post hoc comparison: AD vs CTR p=0.003; AD vs CIND p=0.0001; CIND vs CTR p=0.024).

MCP-1 plasma levels were also valuated in AD patients stratified according to MMSE score. Severe AD (MMSE score <14), showed apparently higher MCP-1 levels (51 pg/ml ±34) than mild AD (41 pg/ml ±27) (MMSE score >14), but differences were not statistically significant.

In addition, MCP-1 plasma levels were correlated with the MCP-1 A-2518G promoter polymorphism. Table 3 shows MCP-1 plasma levels in AD, CIND and controls after the stratification for the three MCP-1 A-2518G different genotypes. It is interesting to note that AD patients and controls carrying AA genotype showed lower MCP-1 plasma levels than CIND (F=7.811 p=0.001). However, MCP-1 genotypes or alleles did not affected MCP-1 plasma levels within each population.

**Table 3:** MCP-1 plasma levels (mean±s.d) in AD patients, CIND and controls

	**AA**	**AG**	**GG**	**Allele A**	**Allele G**	**P**
AD (n=73)	44±32	41±28	39±6	42±29	41±26	0.910
CIND(n=20)	93±33	82±38	66.64±35	86±36	80±37	0.427
CTR(n=15)	44±22	94±113	74.37±50	62±69	91±101	0.697

## Discussion

In AD brain, the presence of amyloid deposition, neurofibrillary tangles, activated microglia and astrocitosis may stimulate a local and a chronic inflammatory process that involves the synthesis and the release of numerous factors such as cytokines, chemokines and inflammatory mediators [13]. However, systemic inflammation is also detectable in peripheral blood of AD as illustrated by the elevation of some acute phase proteins or cytokines [28].

In addition, a large number of genetic variations in genes coding for pro- or anti inflammatory molecules have been associated with the risk of dementia or cognitive decline [4,5,29,30].

In the present study the distribution of MCP-1 A-2518G SNP in the promoter region of MCP-1 gene and plasma levels of MCP-1 in AD or subjects with preclinical AD were investigated.

Our findings suggested that the MCP-1 SNP promoter was not a risk factor for AD being the SNP equally distributed in AD patients and controls. Our results confirmed data from different case-controls studies reporting no association between this SNP and the risk of AD [21,24-26]. On the other hand, a different Italian study by Pola et al. showed an increased representation of GG genotype in AD patients [27]. Our data were not able to confirm this latter study. Differences in SNP distribution across case/controls studies might be explained by the great ethnic variability of this SNP, since the G allele is found differentially present in Caucasian, Americans or Asian population [23]. Differences between present investigation and Pola’s study might be explained by different clinical AD or CTR selection.

Then, we studied the distribution of MCP-1 A-2815G SNP in a MCI population followed up for two years in order to evaluated the effect of this SNP on the progression of the disease. No data on the effect of this SNP in MCI/AD conversion is available in literature. The G allele frequency was slightly higher in MCI showing a further cognitive deterioration and converted to AD. However, this difference was not statistically significant. This effect might be partially ascribed to the small size of MCI population and further investigations are needed to better understand the influence of this SNP in cognitive deterioration and progression to AD.

High levels of pro-(or anti) inflammatory cytokines and chemokines have been found associated with AD [28]. The main role of these molecules and in particular of MCP-1 is supported by the observation that this latter molecule can induce chemotaxis of monocytes and microglia, contributing to pathological gliosis associated with AD [31,32]. Relevance of MCP-1 in AD was suggested by data showing a significantly enhanced immunoreactivity for MCP-1 in neurons, astrocytes and microglia from AD brains [33]. Moreover, several studies reported that MCP-1 plasma or CSF levels were higher in AD patients or MCI than controls [19,20,31].

We found statistically differences in MCP-1 plasma levels among AD, CIND and controls, being MCP-1 levels higher in CIND than those in AD patients and/or controls.

In our study we measured MCP-1 levels by using BIO-Plex multi Cytokines platform. Our results showed that MCP-1 absolute levels, in pg/ml, from AD, CIND or CTR, were lower than those found in other reports. Different techniques for MCP-1 detection may partially explain why we did not confirm increased MCP-1 plasma levels in AD. On the other hand, our data agree with the only study that used the same detection system used by us for MCP-1 levels [12]. Decreased level of peripheral MCP-1 levels along with increased CSF concentration of this chemokyne in AD may suggest an impaired MCP-1 turnover between these two compartments. Such an alteration may be not present in pre dementia conditions such as CIND since, increased levels of blood MCP-1 levels were observed in this population. However, further studies on a larger cohort of CIND or MCI subjects are needed to better define the role of blood MCP-1 in cognitive deterioration and AD developing and the use of blood MCP-1 as a potential marker for cognitive impairment.

MCP-1 expression might be under the control of A-2518G promoter SNP. This is supported by a study showing that the biallelic G/A polymorphism at position 2518 of the MCP-1 gene appears to influence the transcriptional activity MCP-1 gene [22]. In particular G allele seems to be associated with a higher MCP-1 production in a dose dependent manner where GG homozygous produce more MCP-1 than that from G/A heterozygous [22]. A previously reported study showed that MCP-1 serum levels were increased in AD patients positive for one or two G alleles [21]. Our data did not confirm the above findings, since we did not find an increase of MCP-1 levels in AD G carrier patients. Accordingly, no effect of this SNP was observed on MCP-1 plasma levels from CIND or controls.

Our study presents some limitations due to the selection of subject with a different kind of pre-dementia condition: CIND and/or MCI. It is known that MCI, with clinical assessed symptoms and risk factors well described by Petersen RC, are likely to progress to AD at a rate of approximately 12% per year [34]. On the other hand, subjects with CIND show heterogeneous risk factors only partially overlapping with those associated with MCI [35].

Data presented in our pilot study regarding the A-2518G SNP distribution and MCP-1 plasma levels in cognitive deterioration need to be replicated in a homogeneous and larger MCI population to better understand the MCP-1 role in cognitve performance and deterioration.

## Conclusion

Our findings suggest that MCP-1 G allele appears to slightly influence MCI conversion to AD and that MCP-1 plasma levels are increased in preclinical AD such as subjects with CIND. These data implicate that MCP-1 genetic background and phenotypic expression might be associated to early mechanisms associated with neurodegeneration leading to dementia. However, due to the relatively small sample and the different pre-dementia condition subjects included in the present study, our data need to be confirmed in large and homogeneous population-based cohorts.

## Methods

### Subjects

The study included 291 patients with AD (mean age=75.14±7.97), 88 patients with mild cognitive impairment (MCI) (mean age=70.94±8.29), 20 CIND (mean age=78.95±6.47) and 148 healthy subjects (mean age=71.61±4.70 ).

Clinical diagnosis of probable AD was performed according standard clinical procedures and followed the NINCDS/ADRDA [36] and DSM-III-R criteria [37]. Diagnosis of probable AD was performed after patient evaluation by two independent physicians and according brain computerized tomography scan. Cognitive performances and alterations were measured by the mini mental state examination (MMSE).

AD patients were enrolled from Northern Italy at the Department of Neuroscience of Castellanza University (Milan, Italy).

MCI subjects were enrolled from the Department of Neurology, University of Brescia, Northern Italy. Diagnosis of MCI was performed according to current clinical criteria [38].

Subjects with CIND and non demented controls belonged to the “Conselice study of brain aging” [39].

AD patients, MCI and CIND subjects and controls were Caucasian and informed consent from each subject or a relative of each AD patient was obtained.

### DNA extraction and polymorphism detection

DNA extraction from peripheral blood leukocytes were assessed as previously described [40].

The triallelic APOE ε2-4 polymorphism was assessed by a polymerase chain reaction-based method as previously described [41].

The MCP-1 A-2518G promoter polymorphism (rs699947) was detect by PCR reaction, amplification with specific primers: Primer F 5^’^ CCGAGATGTTCCCAGCACAG 3^’^, Primer R 5^’^ CTGCTTTGCTTGTGCCTCTT 3^’^ with 5 min. at 96°C for the initial denaturation and 30 cycles of 1 min. at 96°C, 1 min. at 60°C and 1.5 min. at 72°C. Then, 5 min. incubation at 72°C for final extension was performed. The restriction enzyme PvuII (MBI Fermentas, Italy; 5 U/sample) resolved three different band patterns, according to the three different genotypes; e.g. at 222 bp and 705 bp (GG genotype), 222 bp, 705 bp and 927bp (GA genotype) or only 927 bp (AA genotype). The different bands pattern of the three genotype after agarose gel running, was shown in Figure 1.

### MCP-1 plasma levels measurement

Bio-Plex Cytokine assay (Bio-Rad Laboratories, Hercules, CA, USA) was used to measure MCP-1 plasma levels. Assays were performed following the manufacturer’s instructions (Multi beads assay BioPlex, BioRad). Briefly, a sets of fluorescently dyed beads loaded with capture monoclonal antibodies specific for MCP-1, were used. Plasma samples (50 μl/well of four-fold diluted plasma) or standards were incubated with 50 μl of pre-mixed beads into the wells of a pre-wet 96 well microlitre plate.

After incubation and washing, 25 μl of fluorescent detection antibody mixture were added for 30 min and then the samples were washed and resuspended in assay buffer. High standard curves ranging from 2098.48 to 0.62 pg/ml for MCP-1 was performed as suggested by datasheet. Detection of MCP-1 levels was extrapolated using Bio-Plex Cytokine software.

### Statistical analysis

The Hardy-Weinberg equilibrium was verified for the control group. Two-tailed Pearson’s χ^2^ exact test was used to compare genotype and allele frequencies, the level of statistical significance was set at less than 0.05. A logistic regression model, adjusted for age and APOE ε4 allele was used to evaluate the effect of A-2518G MCP-1 SNP on the risk of AD, the risk of MCI conversion to AD. ANOVA test was used to compare the MCP-1 plasma levels in the tested groups.

## Abbreviations

ACT: Alpha-1 antichymotrypsin; APOE: Apolipoprotein E; IL-1 b: Interlukin-1 beta; IL-6: Interleukin-6; TNFa: Tumor necrosis factor alpha; AD: Alzheimer’s disease; CIND: Cognitive impairment but no dementia; CSF: Cerebro spinal fluid; MCI: Mild cognitive impairment; MCP-1: Monocyte chemoattractant protein-1; MMSE: Mini mental state examination

## Competing interests

The authors declare that they have no competing interests.

## Authors’ contributions

EP, MI, IC performed laboratory analysis and genotyping; MF enrolled AD patients and contributed to the study design; FL coordinated the application of statistical analysis of Conselice data base and contributed to design the clinical, epidemiological and genetic study; FL and EP have been involved in drafting the manuscript. All authors read and approved the final manuscript.
